# A rapid standardized quantitative microfluidic system approach for evaluating human tear proteins

**Published:** 2012-10-12

**Authors:** Piera Versura, Alberto Bavelloni, William Blalock, Michela Fresina, Emilio C. Campos

**Affiliations:** 1 Ophthalmology Unit, Alma Mater Studiorum University of Bologna, Italy; 2 Laboratory of Musculoskeletal Cell Biology-Istituto Ortopedico Rizzoli, Bologna, Italy; 3 CNR-National Research Council of Italy; Institute of Molecular Genetics; Bologna, Italy

## Abstract

**Purpose:**

To explore the potential of a chip-based miniaturized capillary gel electrophoresis device in a quantitative evaluation of the human tear protein profile and to validate the method.

**Methods:**

A total of 5 μl of tears were collected from 25 patients diagnosed as having mild to moderate dry eye according to Dry Eye Workshop guidelines and from 20 matched normal volunteers. Protein analysis was performed with the 2100 Bioanalyzer; different protein kit assays were evaluated (Protein 80 kit, Protein 230 kit, High Sensitivity Protein 250 kit) for sizing and quantifying protein samples from 5 to 80 kDa, 14 to 230 kDa, and 5 to 250 kDa, respectively. A standard protein ladder was loaded on each chip to allow an estimation of the appropriate molecular weight of the separated proteins; a sample buffer containing a lower and an upper marker was used to check the correct alignment of each lane. Virtual bands generated by the Bioanalyzer were identified and validated as follows: tear samples were run in parallel and proteins separated by one-dimensional and two-dimensional sodium dodecyl sulfate–PAGE and characterized by immunoblotting, enzymatic digestion, and analysis with liquid chromatography-mass spectrometry followed by a search of the SProt human protein database.

**Results:**

Analyses were successfully performed by using as small as a 2 μl tear sample. The Protein 230 kit was selected as the best chip kit, able to differentiate all the proteins of interest. The measurement noise parameters were low, and reproducibility and repeatability exhibited high accuracy (0.998 and 0.995, respectively) and precision (0.974 and 0.977, respectively). The coefficient of variability was slightly higher than that declared by the manufacturer (6.2% versus 5.0%). Total protein content and the following proteins were recognized in all samples: lipophilin A lysozyme C, tear lipocalin-1, zinc-alpha-2-glycoprotein, serotransferrin, lactotransferrin, and exudated serum albumin.

**Conclusions:**

Our data demonstrate that this chip-based tear protein analysis is a reliable method of instrumental diagnosis in daily clinical activity and may provide supporting evaluation parameters for diagnosing and managing tear-based disorders.

## Introduction

Tear protein analysis is of increasing interest in ophthalmology [1] since protein content determination has tremendous potential for deepening our knowledge of ocular surface diseases and establishing non-invasive tear-based diagnostic technologies. Human tear proteins have been separated and identified in the past by using various analytical approaches, from the most traditional ones such as monodimensional (1D-GE) [2-6] or bidimensional (2D-GE) sodium dodecyl sulfate–PAGE (SDS–PAGE) [7-9] to more advanced mass-spectrometry techniques [10-13]. The most recent research has been dedicated to identifying novel biomarkers that could provide a protein disease profile, thus assisting with early diagnosis [5,14,15] or monitoring of progression [16,17] in dry eye (DE) disease.

Proteomics is a difficult task in many aspects: due to the enormous complexity of protein mixtures in a biologic fluid, analytical technologies are labor-intensive and sensitive to many processing-related variables, integration of information through bioinformatics is required and is time-consuming, and equipment and consumables are still expensive. Thus, integrating proteomic research into clinical practice is still in progress and has yet to be successful.

To overcome the current challenges in proteomic analysis, new devices have been proposed, based on the developments in electrophoretic separations, where fluids are driven in microstructured channels or capillaries [18-20]. These microchip-based systems provide a great amount of information simultaneously, with a consistent reduction in associated costs, and therefore, they appear a promising tool for application in a clinical setting.

The purpose of the present work was to explore the potential of a chip-based miniaturized capillary gel electrophoresis device in the quantitative evaluation of human tear proteins and to validate the method. To recognize proteins and validate virtual images of gel-like protein profiles from this method, a comparison with profiles obtained with 1D-GE was performed. Bands were characterized with immunoblotting, enzymatic digestion, and mass spectrometry analysis. Human tears from normal subjects and from patients with mild-to-moderate DE were used to recognize and validate the system.

## METHOD

### Subjects

A total of 45 subjects, including 25 patients diagnosed as suffering with mild DE according to a modified Dry Eye Workshop [21] classification (eight men and 17 women; 48.2±8.3. years) and 20 healthy controls (seven men and 13 women; 38.1.±11.8 years) were enrolled; the study was conducted according to the Declaration of Helsinki involving human subjects.

A minimum amount of 5 µl of tears was collected with a laboratory micropipette (Pipetman P, Gilson Int.l B.V., Den Haag, Netherlands) using sterile disposable tips. Briefly, subjects were requested to place their head sideways with the ear touching the back of the reclined chair, for at least 1 min. This position avoids tear drainage from the nose-lachrymal route and allows tears to collect at the lower external canthus where they are then aspirated avoiding any contact and consequently any reflex tearing. Samples were then centrifuged at 13,200 × *g* for 15 min, and the supernatant was aspirated and stored in low protein adsorption surface plastic vials at +4 °C until analysis. On occasion, the storage period was forcibly prolonged up to 2 weeks, in which cases samples were stored at –20 °C.

### Agilent 2100 Bioanalyzer system

Chip-based analysis was performed with the Agilent 2100 Bioanalyzer system (Agilent, Waldbronn, Germany). Preliminary analysis on the most appropriate LabChip kit for our purposes was performed. The Protein 80 kit, the Protein 230 kit, and High Sensitivity Protein 250 kit were tested, for sizing and quantifying protein samples from 5 to 80 kDa, from 14 to 230 kDa, and from 5 to 250 kDa, respectively. All reagents were provided with each LabChip kit, including the standard protein ladder containing different proteins with known concentration and molecular weights that can be used for semiquantitative analysis.

All chips were prepared according to the protocols provided with the individual LabChip kits. Briefly, for the Protein 80 kit and the Protein 230 kit, the samples (1 to 4 μl) were diluted in sample buffer with or without 1 M dithiothreitol solution (DTT). The samples were denaturated by placing the vials in boiling water for 5 min, cooled down afterwards, and centrifuged for 15 s, and then 84 μl deionized water was added to the ladder and samples. A 6 μl aliquot of this solution was loaded onto the chip, which was first filled with a gel/dye mix and destaining solution. Separated proteins were detected with laser-induced fluorescence. The sample buffer used included an upper and a lower marker at known molecular weight.

The sample preparation for the High Sensitivity Protein 250 kit differed only for treatment with solutions containing dimethyl sulfoxide and ethanolamine before boiling. The Agilent 2100 Bioanalyzer system included protein assay analysis software and software for managing patient data.

Commercially purified albumin, lysozyme, lactoferrin (all from Sigma), and recombinant lipophilin A and C (courtesy gift from Dr. Joerg Klug, Institut fuer Anatomie und Zellbiologie, JLU Giessen, Germany) and lipocalin (courtesy gift from Prof. Bernhard Redl, Division of Molecular Biology Biocenter-Innsbruck Medical University, Austria) were run as standards to observe and evaluate their separation and migration patterns.

### Monodimensional electrophoresis

Tear proteins were separated by using an 18% acrylamide Tris-HCl Ready Gel (Bio-Rad, Laboratories Inc., Hercules, CA) applying a voltage of 200 V for 1 h at room temperature (r.t.), as detailed elsewhere [17]. Briefly, samples were diluted 1:2 in 0.125 M Tris-Cl, pH 6.8, 4% sodium dodecyl sulfate (SDS), 20% glycerol, and 10% 2-mercaptoethanol, and boiled for 5 min.

Gels were stained with Brilliant Blue G (Sigma) for 12 h at r.t. and then thoroughly washed with 25% methanol in distilled water.

Gel images were acquired with the densitometer scanner Umax (Amersham Biosciences-GE Healthcare, Milan, Italy) and analyzed for the percent abundance of each protein of interest in the samples using Gel-Pro Analyzer software (MediaCybernetics Inc., Bethesda, MD).

A calibration curve was constructed by using albumin, lysozyme, and lactoferrin purified reagents (Sigma), diluted to prepare graded standard solutions at different concentrations (3.0, 1.5, and 0.75 mg/ml).

### Total tear protein

The total protein content in each tear sample was measured by using a Bradford’s based enzyme-linked immunoassay (ELISA) kit (Bio-Rad) according to the manufacturer’s instructions.

### Immunoblotting

The proteins from 1 µl of tears were subjected to SDS–PAGE at a constant 200 V using a Mini-Protean III (Bio-Rad) and transferred onto nitrocellulose membrane (Hybond-C Extra, Amersham) applying a voltage of 100 V (1 h, 4 °C) in buffer containing 0.3% Tris, 1.4% glycine, and 20% methanol using a Bio-Rad wet-blotting apparatus. The nitrocellulose membrane containing the transferred proteins was saturated with 3% BSA (Sigma) in phosphate saline buffer (PBS) + 0.1% Tween-20 for 1 h at room temperature. For immunoblotting the antialbumin (H-126; sc-50535), antitransferrin (M-70; sc-30159), antilactoferrin polyclonal antibody (sc-25622), antilipocalin (sc-34680), antilysozyme (sc-27956), antilipophilin A (sc-48324), and antilipophilin B (sc-48327) were purchased from Santa Cruz Biotechnology. The anti-zinc-alpha-2-glycoprotein (ZAG) rabbit polyclonal antibody was purchased from BioVendor GmbH (Heidelberg, Germany).

Primary antibodies were diluted in PBS containing 0.1% Tween-20 and incubated overnight at 4 °C. The blots were washed three times with PBS + 0.1% Tween-20 and incubated with horseradish peroxidase–conjugated secondary antibodies diluted (1:10000) in PBS + 0.1% Tween-20 for 1 h at room temperature. All membranes were revealed using an electrochemiluminescent system (Supersignal West Dura Extended Duration Substrate, Pierce Biotechnology Corp., Rockford, IL).

### Bidimensional electrophoresis

One μl of tears was used for the procedure. For the first electrophoretic dimension 7 cm strips (pH 3–10) were used (Bio-Rad). The active rehydratation of the strip with the sample was performed at 50V for 16 h, followed by step increase in voltage of 1000V (1 h), 2000V (1 h), 4000V (2h), and 4000V (20 kV-h in total). The strips were then reduced using equilibration buffer (6M urea, 2% SDS, 5 mM Tris-HCl pH 8.6, 30% glycerol, containing 125 mM DTT, for 15 min at room temperature and alkylated with equilibration buffer containing 250 mM iodoacetamide (IAA) for 8 min. The strips were transferred to a 15%-polyacrylamide gel, and, for the electrophoretic separation,a Mini-Protean III apparatus (Bio-Rad) with a constant voltage (200V) was used. The proteins were stained with colloidal Coomassie Blue G-250 (Sigma) for 12 h at room temperature.

### In-gel tryptic digestion

The 1D and 2D electrophoresis gel excised slices or spots containing the proteins of interest were reduced with 10 mM DTT for 45 min at 56 °C, alkylated with 55 mM iodacetamide for 30 min at room temperature in the dark, and incubated overnight at 37 °C in a 50:1 (w/w) ratio with 12 ng/µl sequencing-grade-modified trypsin (Promega, Madison, WI). The digested peptides were extracted from the slices by adding water and 5% trifluoroacetic acid and 50% acetonitrile. Peptides were lyophilized to dryness and resuspended with 10 µl of 0.1% formic acid for mass spectrometry analysis.

### Mass spectrometry analysis and database search

The samples were analyzed with liquid chromatography mass spectrometry (LC-MS) using a CapLC (Waters, Manchester, UK), connected with an electrospray interface quadrupole-time-of-flight (ESI-Q-TOF) micromass spectrometer (Micromass, Manchester, UK). The peptide separation was performed on an Atlantis dC18 NanoEase column (150×0.3 mm, 3 µm; Waters) with an Atlantis dC18 NanoEase precolumn (5×0.3 mm, 5 µm particle size; Waters) with a flow rate of 4 µl/min (mobile phase A: H_2_O/acetonitrile (95:5) 0.1% FA; B: acetonitrile/H_2_O (95:5) 0.1% FA). The chromatographic gradient was set up to give a linear increase from 2% B to 80% B in 30 min, for a total run-time length of 45 min. For the identification experiments, the quadrupole-time-of-flight mass spectrometer was set to scan in survey mode in the m/z 400–1800 range.

For protein identification, Mascot (version 2.02.03) and the Swiss-Prot human database (version 52.2, 495,929 in total) were used, setting a 50 ppm precursor and 0.3 Da fragment tolerance, carbamidomethylation of cysteine as fixed modification, oxidation of methionine as variable modification, and trypsin as enzyme (one miss cleavage allowed).

## Results

Preliminary experiments were performed to establish standardized conditions that could yield reproducible results for the following parameters: preanalytical sample storage, dilution, temperature, and reducing conditions. Modifications in the storage period (range from 2 h to 2 weeks) and temperature (4 °C, −20 °C, −80 °C) did not significantly change either the protein profile or the protein quantification. Satisfactory protein separation was achieved by boiling the samples for 5 min and including DTT in the sample buffer. To obtain a well defined protein separation and a readable virtual band image, a 2 μl sample was the most suitable amount of tears for analysis in the system.

**Figure 1:**
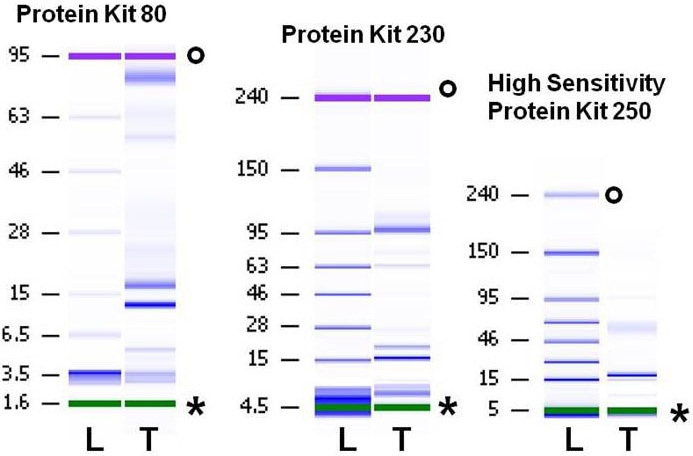
Tear protein profile of the same sample (normal subject) run with different LabChip kits; the Protein 80 kit, the Protein 230 kit, and the High Sensitivity Protein 250 kit. On the left of each lane are the markers specific for each LabChip kit along with the corresponding molecular weight values. **L**:=ladder, **T**:=tear sample, degree symbol=upper marker, *=lower marker.

The standard protein ladder was loaded on each chip and analyzed as the first sample (Figure 1, lane L), thus allowing for the correct alignment of each lane and an estimation of the appropriate molecular weight of the separated proteins (Figure 1, lane T). The sample buffer contained a lower and an upper marker (Figure 1 marked with _*_ and _°_), different for each LabChip kit used: 5 kDa and 80 kDa for the Protein 80 kit, 14 kDa and 230 kDa for the Protein 230 kit, and 10 kDa and 250 kDa for the High Sensitivity Protein 250 kit.

**Figure 2:**
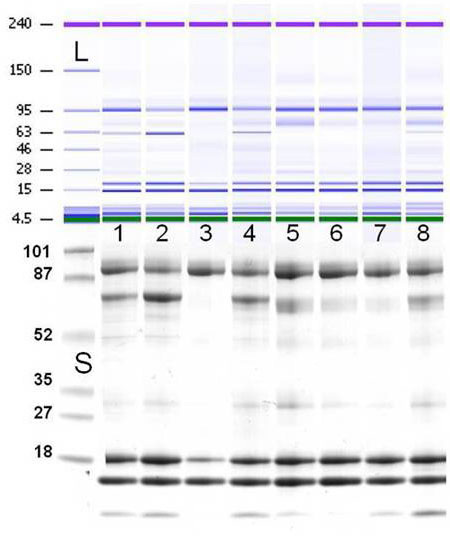
Analysis of tear proteins obtained by running aliquots from the same samples in parallel. Tear samples (one to eight) were runned through the 2100 Bioanalyzer (upper part of the figure, Protein 230 kit, gel-like view), and the 1D SDS–PAGE (lower part of the figure). The corresponding molecular weight in kDa is reported on the left of the ladder **L**: and of the prestained protein standard **S**: The same profiles were obtained for each sample in the two analytical methods.

Protein profiles obtained by running aliquots from the same sample in parallel through the SDS-Gel and the 2100 Bioanalyzer did not substantially differ in bands (Figure 2). The total tear protein amount obtained by running the same sample through the 2100 Bioanalyzer and the ELISA system did not show a significant difference (p always >0.05). The total tear protein content did not show any significant difference dependent upon the type of LabChip kit used.

**Figure 3:**
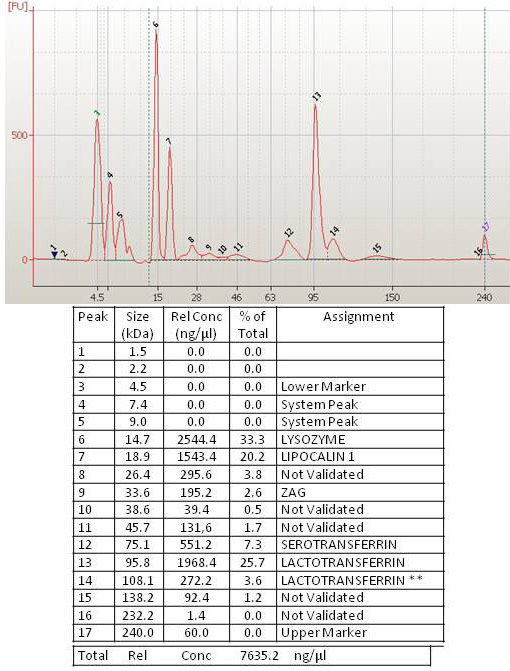
In the upper part is the electropherogram view of a normal tear sample (also run on lane 6 of Figure 2), the molecular weight in kDa is on the x-axis, and the fluorescent intensity is on the y-axis. Peaks are identified for all samples and are tabulated by peak ID (numbers 1 to 17 at the top of each peak) in the customizable result table shown in the lower part. In this table, the size in kDa, the concentration in ng/μl, and the percentage versus total protein content for each peak are shown. **A shoulder peak for lactotransferrin is found in this analysis performed with the Protein 230 kit.

**Figure 4:**
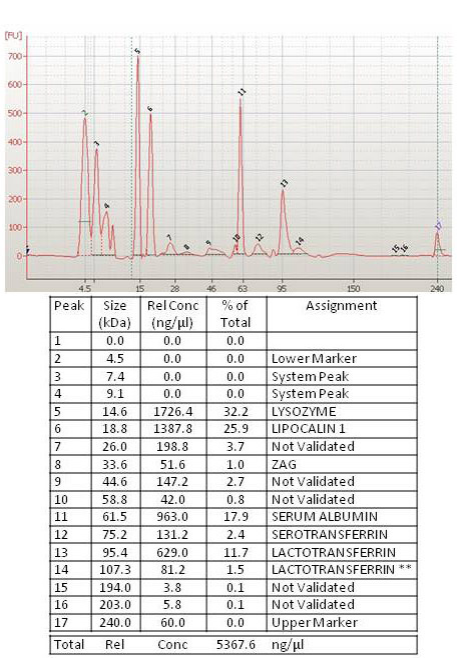
In the upper part is the electropherogram view of the tear sample from a patient with dry eye (also run on lane 2 of Figure 2), the molecular weight in kDa is on the x-axis, and the fluorescent intensity is on the y-axis. Peaks are identified for all samples and are tabulated by peak ID (numbers 1 to 17 at the top of each peak) in the customizable result table shown in the lower part. In this table, the size in kDa, the concentration in ng/μl, and the percentage versus total protein content for each peak are shown. **A shoulder peak for lactotransferrin is found in this analysis performed with the Protein 230 kit.

The 2100 Bioanalyzer software can convert each protein band and intensity parameters into sizing peaks, to obtain separate electropherograms from virtual band images. Figure 3 shows an example electropherogram from a tear sample of a normal subject and Figure 4 from a tear sample of a patient with DE. The Bioanalyzer identified differences between pathological and normal samples. In the lower part of the figures, data emerging from analysis are summarized, with an explanation in the legends. We did not automatically assign a protein identity to each band or peak detected, since the molecular weight (kDa) of a protein derived from the 2100 Bioanalyzer did not always precisely correspond to the established kDa reported in protein databases.

Characterization of virtual bands through proteomic techniques applied to the SDS gels electrophoresed in parallel from the same samples demonstrated the constant and reproducible detection of seven proteins, which were present in tears either from normal subjects or patients with dry eye. Results of the protein identification by mass spectrometry and database search are shown in Table 1 and refer to the major proteins expressed in all samples. In particular, lysozyme C (LYS-C), tear lipocalin-1 (LIPOC-1), zinc-alpha-2-glycoprotein (ZAG-2), serotransferrin (TRANSF), lactotransferrin (LACTO), and exudated serum albumin (ALB) were recognized. Comparative evaluations among the available kits demonstrated that the Protein 230 kit exhibited the most suitable range for simultaneously detecting these proteins and represented the best option for routine practice. Lipophilin A (LIPOPH-A) was detected in all samples only in the profiles obtained with the Protein 80 kit.

**Table 1:** Results of the protein identification by mass spectrometry and database search.

Protein name	Entry name	Protein score	Protein mass (Da)	# of identifed peptides	Protein coverage (%)
Lactotransferrin	TRFL_HUMAN	737	80,014	14	22
Serotransferrin	TRFE_HUMAN	44	79,280	5	3
Serum albumin	ALBU_HUMAN	698	71,317	21	40
Zinc- α-2-glycoprotein	ZA2G_HUMAN	34	34,079	3	5
Lipocalin-1	LCN1_HUMAN	242	19,409	9	29
Lysozyme C	LYSC_HUMAN	108	16,982	8	42
Lipophilin A	SG1D1_HUMAN	86	10,234	3	15

The molecular weight calculated to the protein ladder for each tested Lab-chip are summarized in Table 2. In particular, the detection system can calculate each individual protein percentage as a function of the total protein detected for each individual run. A molecular weight range is given due to the small migration and injection variables observed from tear sample to tear sample.

**Table 2:** Tear proteins recognized by 2100 Agilent Bioanalyzer and validated in the study, listed along with their name and code from S-Prot human database, theoretical molecular weight in kDa and function.

Protein name	Protein name database	Theoretical size (kDa)	1D SDS–PAGE size (kDa)	Bioanalyzer size range (kDa) Lab-chip Kit			Tear/ plasma derived	Function
				80	230	HS 250		
Lysozyme	LYSC_HUMAN (P61626)	14	13.8–14.5	12.8–13.7	14.3–15.0	14.8–15.1	Tear	Antibacterial enzyme; destroys the cell walls of certain bacteria. Innate immunity
Lipocalin-1	LCN1_HUMAN (P31025)	17	16.5–17.6	16.7–18.4	18.1–19.9	17.7–18.8	Tear	Ability to bind small, hydrophobic molecules such as retinol to transport and protect
Zinc-a2-glycoprotein (ZAG)	ZA2G_HUMAN (P25311)	34.2	33.0–35.0	21.5–24.6	30.5–36.0	29.7–35.6	Tear, plasma	It stimulates lipid breakdown in adipocytes, it may have a role in the expression of the immune response, specific role of ZAG in tears not known
Albumin	ALBU_HUMAN (P02768)	68	65.0–68.0	56.7–57.6	59.1–65.4	57.1–58.5	Plasma	Transportation of free fatty acids, stabilizing the osmotic pressure
Lactotransferrin	TRFL_HUMAN (P02788)	82	78.0–83.0	83.0–84.2	93.7–99.3 105.2–110*	96.5–99.1	Tear	Tear Inhibitor of bacterial growth possible anti-inflammatory properties. Innate immunity
Serotransferrin	TRFE_HUMAN (P02787)	77	70.0–72.0	64.8–65.1	73.5–78.4	86.0–89.2	Plasma	Iron binding transport proteins, expressed by the liver and secreted in plasma
Lipophillin A	SG1D1_HUMAN (O95968)	9.8	8.9–9.2	5.2–5.3	9.3–11.0	12.8–13.0	Tear	May bind androgens and other steroids, possibly under transcriptional regulation of steroid hormones.

The kDa size for each protein is also reported for 1D_SDS–PAGE electrophoresis and for the Bioanalyzer, in this last case for all the three types of Lab-chip kit evaluated in the study. For both methods, a molecular weight range in kDa is given due to small migration and injection variables observed from tear sample to tear sample. * A shoulder peak found in purified standard lactoferrin and in all samples, shown only with P230 Lab-chip kit.

**Figure 5:**
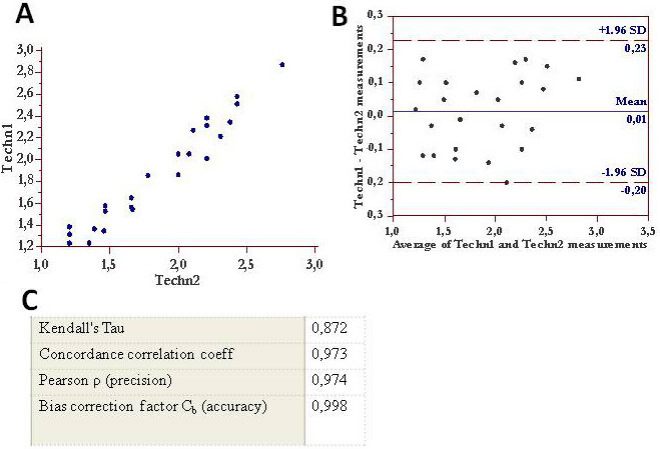
Inter-practitioner (Techn1 and Techn2) measurement variability (reproducibility) for the Agilent 2100 Bioanalyzer is shown. **A**: The tear lysozyme measurements (mg/ml) performed by Techn1 (y-axis) and by Techn 2 (x-axis) on the same tear samples are graphed. **B**: The Bland–Altman plot for various tear samples analyzed for lysozyme content, performed by two different laboratory technicians (Techn 1 and Techn 2), is shown. The x-axis indicates the mean of the lysozyme content in mg/ml obtained by Techn 1 and Techn 2. The y-axis indicates the difference in measurement between the two technicians. The mean difference of the technicians is 0.01, with an upper specification limit of 0.23 and a lower specification limit of 0.20. **C**: The concordance correlation coefficients calculated for the two technicians are shown; all values demonstrated the high reproducibility of the Bioanalyzer method.

**Figure 6:**
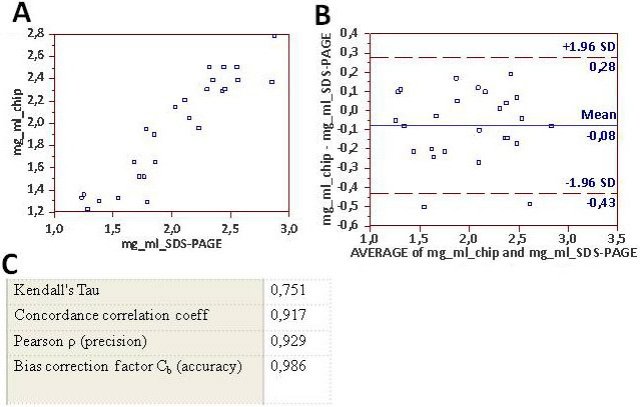
Degree of agreement between measurements conducted on replicate specimens. The same tear samples were runned with two methods: monodimensional sodium dodecyl sulfate-polyacrylamide lectrophoresis (1D SDS–PAGE) electrophoresis and the 2100 Agilent Bioanalyzer (Protein 230 kit). **A**: The measurements obtained with 1D-SDS–PAGE electrophoresis (y-axis) and the 2100 Agilent Bioanalyzer (x-axis) are graphed. **B**: The Bland–Altman plot for various samples of tears analyzed for lysozyme content, performed with the two methods, is shown. The x-axis indicates the mean of the lysozyme content in mg/ml obtained with 1D SDS–PAGE electrophoresis and the 2100 Agilent Bioanalyzer. The y-axis indicates the difference in measurement between the two methods. The mean difference is 0.08, with an upper specification limit of 0.28 and a lower specification limit of 0.43. **C**: The concordance correlation coefficients calculated for the two methods are shown; all values demonstrated high correlation between the two methods.

**Figure 7:**
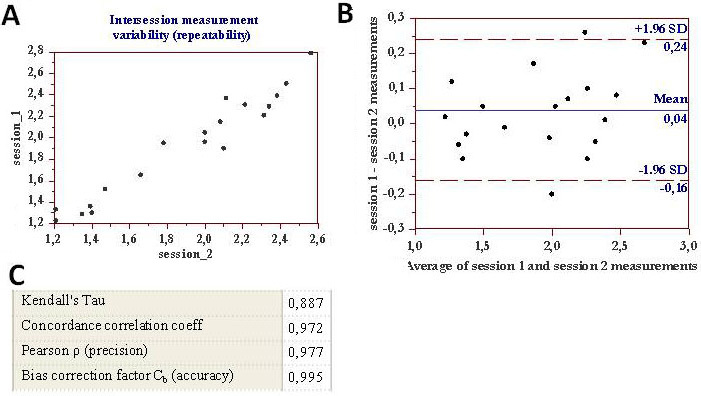
Inter-session (session 1 and session 2) measurement variability (repeatability) for the Agilent 2100 Bioanalyzer is shown. **A**: The tear lysozyme measurements (mg/ml) performed during session 1 (y-axis) and session 2 (x-axis) on the same tear samples are graphed. **B**: The Bland–Altman plot for various tear samples analyzed for lysozyme content, performed in two sessions, is shown. The x-axis indicates the mean of the lysozyme content in mg/ml obtained in session 1 and session 2. The y-axis indicates the difference in measurement between the two sessions. The mean difference of both sessions is 0.04, with an upper specification limit of 0.24 and a lower specification limit of 0.16. **C**: The concordance correlation coefficients calculated for the two sessions are shown; all values demonstrated the high repeatability of the Bioanalyzer method.

Calculation of measurement noise for Agilent 2100 Bioanalyzer applied to detecting tear proteins exhibited a high degree of precision and accuracy in terms of reproducibility (Figure 5 and Figure 6) and repeatability (Figure 7) . A good correlation coefficient was also found for total tear protein content between measurement performed with the ELISA technique and 2100 Agilent Bioanalyzer (Kendall's Tau 0.903; Concordance correlation coefficient 0.965).

The amount of time used for analysis was 40 min for the preanalytical process for the samples and 30 min for analysis, for 10 samples altogether run in a single chip. The estimated cost for each sample was estimated at €3.50, including reagents and labor costs.

## Discussion

The 2100 Bioanalyzer, together with the Protein 230 kit, provided validated data for the total tear protein content and concentration of specific tear proteins in pathological and normal samples with significant time savings, cost reduction, improved ease of use, and high reproducibility compared to traditional 1D SDS–PAGE. The equipment, also referred to as a “lab-on-chip” device, offers the advantage of quantitatively estimating multiple protein species without dividing the original patient sample, which is often limited in the case of patients with DE.

Previous papers had postulated the impact of this application in clinical routine [22,23], but to our knowledge, this is the first attempt to validate the method in analyzing the protein composition in pathological samples, demonstrating the diagnostic potential of this method. The system consists of a miniaturized capillary gel electrophoresis device integrating all steps of a “conventional” electrophoretic run, from sample preparation, labeling, gel loading, and separation up to detection with a laser-induced fluorescence detector.

One major hindrance relates to the protein sizing assay since the expected theoretical and observed molecular weights may and actually do differ to some extent. The reasons for this can only be theorized. As Mann and Tighe [22] have discussed, the differences may be possible consequences of protein denaturation, post-translational modification, injection efficiency, sample salt content, or injection variables. However, in 1D SDS–PAGE a degree of shift in the theoretical molecular weight may also occur depending upon the sample preanalytical preparation and post-translational modification.

To validate data from the 2100 Bioanalyzer and kits, proteomic recognition of selected protein bands was performed on samples run in parallel on 1D SDS–PAGE and by analyzing the electrophoretic behavior of purified proteins run in adjacent lanes of the chip. Our study demonstrated and validated the presence of lipophilin A-C, lysozyme-C, tear lipocalin 1, zinc-alpha-2-glycoprotein, serotransferrin, lactotransferrin, and exudated serum albumin.

Tear analysis performed with the 2100 Bioanalyzer and Protein 230 kit showed more distinct peaks compared to the 1D SDS–PAGE. In particular, transferrin was recognized distinctively in all samples, and lactotransferrin appeared as a major and a shoulder peak, in agreement with what was found by previous authors using another LabChip kit no longer commercially available [22]. Expanding the validation of the remaining proteins is relevant to advancing basic research and clinical medicine, and it is a future aim.

However, we succeeded in creating a panel of proteins assay of clinical significance for ophthalmologists that can aid in diagnosing and stratifying mild or early DE. The Bioanalyzer identified differences between pathological and normal samples for all the specific proteins recognized and in the total protein amount. However, the purpose of the present paper was to validate the method, and we did not go into too much detail in analyzing the clinical impact; this will be the focus of a further step, now in advanced finalization. We preferred to present and discuss the data separately, to enhance the relevance of the technical validation data (a huge amount of “preliminary” work) and the clinical application section.

At this stage, we demonstrated that the panel of tear proteins recognized with this laboratory-on-chip approach could also be of interest and applicable in screening or monitoring disease to better understand the pathophysiology of a particular disease process. As a conclusive comment, this microfluidic approach applied to the tear proteome can provide validated data for trials investigating clinical or therapeutical outcomes and is suitable for affordable application in a clinical setting, in terms of time and costs. This approach can be applied in daily laboratory activity following a minimal period of training of personnel.
